# Using Scent Detection Dogs in Conservation Settings: A Review of Scientific Literature Regarding Their Selection

**DOI:** 10.3389/fvets.2016.00096

**Published:** 2016-10-28

**Authors:** Sarah C. Beebe, Tiffani J. Howell, Pauleen C. Bennett

**Affiliations:** ^1^Anthrozoology Research Group, School of Psychology and Public Health, La Trobe University, Bendigo, VIC, Australia

**Keywords:** conservation, detection dog, olfaction, scent, biopsychosocial, scat, wildlife, selection

## Abstract

Dogs are widely used for scent detection work, assisting in searches for, among other things, missing persons, explosives, and even cancers. They are also increasingly used in conservation settings, being deployed for a range of diverse purposes. Although scent detecting dogs have been used in conservation roles for over 100 years, it is only recently that the scientific literature has begun to document their effectiveness and, importantly, how suitable dogs should initially be selected by organizations wanting to develop a detection program. In this paper, we review this literature, with the aim of extracting information that might be of value to conservation groups considering whether to invest in the use of dogs. We conclude that selection of appropriate dogs is no easy task. While olfactory ability is critical, so also are a range of other characteristics. These include biological, psychological, and social traits. At present, no validated selection tools have been published. Existing organizations have adapted selection instruments from other contexts for their use, but very little published information is available regarding the effectiveness of these instruments in a conservation setting. In the absence of clear guidelines, we urge those wanting to invest in one or more dogs for conservation purposes to proceed with extreme caution and, preferably, under the watchful eyes of an experienced professional.

## Introduction

The use of dogs in conservation detection first emerged in the 1890s, when dogs were successfully used to locate the New Zealand kiwi (*Apteryx* spp.) and kakapo (*Strigops habroptilus*) (1, 2). Up until the early 1990s, conservation detection dogs (CDDs) focused predominantly on detection of live birds [(3); see Ref. (4) for additional references]. Recent years, however, have seen a rapid expansion of the field. Conservation detection now encompasses an array of activities, including detection of live wildlife (5–8), carcass detection for birds and bats around wind turbines (9–12), and detection of scats, pathogens, and other biological materials (13–16). Several reports indicate that, in many cases, CDDs are more efficient than several other survey methods in detecting the presence/absence, and relative abundance, of plants and wildlife (3, 10, 15, 17, 18). These animals therefore represent an exciting opportunity which could substantially benefit conservation groups worldwide. However, the costly nature of selecting, training, and housing CDDs (2), and uncertainty regarding why some individual dogs succeed in tasks where others perform poorly (19, 20), may act as barriers preventing more widespread use.

In order to improve selection efficiency in other dog-based scent detection contexts, relevant organizations have developed comprehensive assessment tools. Not many of these are publicly accessible, although a few have been described (7, 14). For example, the Brownell–Marsolais scale (21), used with search and rescue (SAR) and disaster dogs, reportedly allows one to measure pack, food, and play drives, as well as motivation and nerve strength, in scent detection dog candidates. To the best of our knowledge, no such tools exist for CDD selection. Organizations selecting and assessing potential CDDs have reported adapting assessment instruments from other fields, but it seems likely that conservation detection has unique requirements, which might mean that existing tools do not capture the full catalog of required traits. They are also difficult for inexperienced persons to acquire or implement effectively.

A dearth of publicly available knowledge regarding how to select relevant dogs is potentially a significant barrier for organizations wanting to use scent detection dogs in conservation settings. Conservation groups often involve volunteers, and they are typically small, local organizations with access to limited expertise and resources, unlike police and military detection units, which are comparatively well resourced. Even larger organizations employing dogs for conservation purposes tend to source their dogs from shelters or from among those owned by volunteers and may benefit from access to information about successful selection practices used elsewhere. Hurt and Smith (22) reported that, of potential CDDs sourced from shelters, as few as one in every 200–300 dogs may be selected. Of these, only 40% may successfully complete the training program.

Low training-completion rates undoubtedly reflect many factors that intervene between selection and eventual certification, including health and performance issues not related to initial suitability. Nonetheless, there is a clear need for development of standardized tools, which may be deployed reliably throughout the entire industry. Experienced handlers typically report preferring to work with dogs with high energy and strong motivational drives, which may make them unsuitable as family pets, yet conservation groups may require their dogs to live primarily as pets. The dogs may also perform their conservation role infrequently, in fragile ecosystems, some containing critically endangered plants or animals, and in challenging search environments where success may be greatly affected by environmental conditions, such as wind direction and strength. It is therefore imperative that these dogs are highly responsive to their human handlers and able to perform consistently across trials regardless of challenges provided by local conditions.

A key benefit of established selection tools in other contexts is that they assess several different characteristics believed to be relevant to scent detection dog success. Similarly, we propose that, in the case of CDDs, a multidimensional model may provide a useful conceptual framework for informing development of selection tools. For this reason, we advocate use of the biopsychosocial (BPS) model, first developed as a tool for psychiatric medicine by Engel (23, 24). Engel (23) argued that the prevailing biomedical model was insufficient to account for many medical problems and for patient outcomes following treatment. Instead, he asserted, these outcomes typically reflect a combination of biological characteristics (genetic predispositions and physiological mechanisms), psychological processes (perceptions, beliefs, attitudes, personality, and attachment), and social contexts (social structure, cultural influences, and other interpersonal relationships).

The BPS model has widespread appeal because it reminds us to consider whole systems, assuming from the outset that most behavioral outcomes will reflect a complex combination of factors, some of which may interact in unexpected ways (25–27). With regards to scent detection dogs, it seems clear that many dog characteristics are relevant. Moreover, each dog’s characteristics are likely to interact with those of its trainer, handler, and environment to determine overall success. One of the main advantages of a BPS approach, however, is that the system can operate on many levels. Analysis at one level (e.g., dog) can therefore be used to improve understanding at a higher level (e.g., dog-handler team). With that in mind, the aim in this review is to examine the importance of biological, psychological, and social characteristics in selection of candidates for training as CDDs.

## Literature Search

First, we conducted a literature search using combinations of key words from among the following list: BPS, working dog, scent detection, wildlife detection, scat detection, carcass detection, breed, temperament, personality, behavior, social intelligence, and assessment tools. Sources searched included the Google Scholar, Science Direct, and Web of Science databases. Second, we examined the reference lists of studies from our first search in order to find relevant studies we may have missed. Finally, we searched reports from individual CDD organizations to determine whether more information was available on selection and training in cases where sufficient information was not provided in the published scientific literature. We compiled a list of studies which provided sufficient detail regarding the dogs used, selection, training and search methods employed, and accuracy/efficiency of CDD teams. Of the more than sixty studies identified, we selected 30 studies as a sample of available information (Table 1). These 30 studies where chosen in order to represent the broad diversity of search roles and environments in which CDDs are often employed.

**Table 1:** **Summary of a sample of wildlife, carcass, scat, and nest detection studies and the techniques used/characteristics selected for when assessing dogs as potential conservation detection dogs (CDDs)**.

Reference	Target	Dog-handler teams	Accuracy/efficiency	Selection and training based on
Arandjelovic et al. (28)	Scat detection: lowland gorilla	3 CDDs; >1 year experience	Dogs detected 43 fresh and 288 old scat samples in 44 days	Selection: professional CDD organization
			Fresh scats were more likely to be detected by dogs (72%) than humans (39%)	Reward: no mention
				Training: authors refer to published CDD literature
Brook et al. (29)	Scat detection: Javan rhinoceros	2 CDDs; >1 year experience	Dogs detected 22 scats over 118 days, and ~429 km	Selection: professional CDD organization
				Reward: no mention
				Training: professional CDD organization
Browne et al. (30)	Scat and skin detection: 3 species of reptile	20 CDDs; <1 year experience; 8 Retrievers, 2 Shepherds, 1 Spaniel, 5 working dog mixes, 4 non-working breeds	Average success by dogs to detect Tuatara (i) scent was 85.0%, (ii) scats was 97.8%, and (iii) skin was 95.6%	Selection: experience in competitive obedience scent discrimination exercises
			Average success by dogs to detect Gecko (i) scent was 77.8%, (ii) scats was 77.8%, and (iii) skin was 51.8% (Detection rates for gecko increased with repeated trials)	Reward: no mention
				Training: standard scent obedience exercises
Cablk and Heaton (31)	Burrow and live reptile detection: desert tortoise	2 CDDs; no information	Accuracy for nest detection by dogs was 90%	Selection: play drive, low–moderate hunt drive, direction and control with handler
			Efficiency between dogs and humans was similar to find live tortoises	Reward: play
				Training: described in article
Chambers et al. (32)	Roost and scat detection: bat	2CDDs; experienced	Accuracy of dogs to detect bat scats at 6 m above ground was 20% and at 2 m above ground was 60%	Selection: professional CDD organization
				Reward: no mention
				Training: professional CDD organization
Cristescu et al. (33)	Scat detection: koala	1 CDD; >1 year experience; Collie	Dogs were 153% more accurate than human surveyors	Selection: ball drive and motivation
			Dogs were 19 times faster than human surveyors	Reward: play
				Training: professional dog trainer
Dematteo et al. (18)	Scat and burrow detection: bush dog	1 CDD; experienced	Dogs detected 11 dens over 72 days and 218.4 km	Selection: professional CDD organization. Also mentioned were body type (robust) and disposition (no territorial behavior)
				Reward: play
				Training: professional CDD organization
Duggan et al. (8)	Live animal detection: Franklin’s ground squirrel	2 CDDs; experienced	Accuracy of dogs and human surveyors was similar at 83 and 84%, respectively	Selection: professional CDD organization. Also mentioned were object obsession and play drive
			Efficiency of dogs was 10 times faster than humans	Reward: play
				Training: professional CDD organization. Authors also refer to published CDD literature
Hagell (34)	Scat detection: spider monkey	1 CDD; experienced	Dogs and human surveyors scored similarly in comparison trials. However dogs were less accurate (59%) than humans (82%)	Selection: professional CDD organization
				Reward: no mention
				Training: authors refer to published CDD literature
Harrison (17)	Scat detection: bobcat	1 CDD; no information	Dogs were over 10 times more effective in detecting bobcats than scent traps, camera traps, and hair scares combined	Selection: professional CDD organization
				Reward: play
				Training: professional CDD organization
Kerley and Salkina (19)	Scat discrimination: Amur tiger	5 CDDs; experienced; 1 Shepherd, 1 Pointer, 3 mixed breeds	Accuracy of scat discrimination increased from 87 to 98% with repeated trials in 4 of the 5 dogs	Selection: play/food drive of pups and their parents
				Reward: play/food
				Training: authors refer to published CDD and scent discrimination literature
Leigh and Dominick (16)	Scat detection: spotted tailed quoll	1 CDD; <1 year experience; Shepherd	Accuracy of dogs in grassland and heath was 83% and in woodland was 87%	Selection: professional CDD organization. Also mentioned were play drive, temperament, high energy and intelligence. Authors also refer to published CDD literature
				Reward: play
				Training: authors refer to published CDD and scent-detection literature
Long et al. (6)	Scat detection: 3 forest carnivores	5 CDDs; experienced	1596 scats found over 2 years (~3.6 scats/km). Of these, 83% were unlikely to be found without CDDs	Selection: professional CDD organization. Also mentioned were drive/object orientation and appropriate temperament
				Reward: play
				Training: professional CDD organization. Authors also refer to published CDD literature
Mathews et al. (12)	Carcass detection: bat	2 CDDs; <1 year experience; 2 Retrievers	Accuracy of dogs was 75%, which was significantly higher than human surveyors who detected 20% of bat carcasses	Selection: ball drive (search and play)
			Efficiency of dogs over 4 times faster than humans	Reward: play
				Training: trained by experienced police dog handlers
Nussear et al. (7)	Live reptile detection: desert tortoise	6 CDDs; varying experience; 1 Collie, 2 Shepherds, 1 Kelpie, 2 Retrievers	Accuracy was similar between human and dog teams of ~70%. Dogs found more targets under vegetation than humans	Selection: drive, previous scent experience, and training
				Reward: no mention
				Training: authors refer to published CDD literature
O’Connor et al. (20)	Nest searches: bumble bee	1 CDD; <1 year experience; Spaniel	Accuracy of dog to detect known bumble bee nests was 62.5%	Selection: authors refer to published CDD literature
			In uncontrolled test dog performed similar to human surveyors (CDD 1.41 nests/ha; humans 1.44 nests/ha)	Reward: no mention
				Training: authors refer to published CDD literature
Oliveira et al. (35)	Scat detection: *Mazama* deer	1 CDD; <1 year experience; mixed breed	Dog detected 8 scat samples across 39 km of trails searched, approximately 0.21 samples/km. Human surveyors detected no scat samples, but did record 24 deer tracks	Selection: professional organization
				Reward: play
				Training: military police and narcotics detection dogs
Paula et al. (11)	Carcass detection: bird	1 CDD; <1 year experience; 1 Shepherd	Accuracy of dog was 96%, which was significantly higher than human surveyors who detected 9% of bird carcasses	Selection: object orientation, high drive, and appropriate temperament
				Reward: play
				Training: authors refer to published CDD literature
Reed et al. (36)	Scat detection: 6 carnivores	2 CDDs; <1 year experience; 2 mixed breeds	Accuracy decreased in dogs with increasing detection distance. At 10 m from transect, dog detection rates were >75%	Selection: professional CDD organization. Also mentioned were object obsession and agility. Authors also refer to published CDD and scent detection literature
				Reward: play
				Training: professional CDD organization. Authors refer to published CDD literature
Reindl (37)	Scat detection: black-footed ferret	2 CDDs; no information	Accuracy of dogs was 86% for areas of known ferret populations	Selection: professional CDD organization. Also mentioned were play/food drive and focus, consistent concentration, and agility
				Reward: play/food
				Training: professional CDD organization (details provided in text)
Robertson and Fraser (38)	Live bird detection: kiwi and kakapo	3 CDDs; experienced; 2 Retrievers, 1 Setter	Of radio-tagged birds, dogs detected 36% adult kiwis and 24% sub-adults	Selection: temperament test by professional organization at 6–12 months into training
				Reward: no mention
				Training: described in article
Rolland et al. (39)	Scat detection: right whale	2 CDDs; no information	Detection by dogs (*n* = 97 scats) was more successful than humans (*n* = 30 scats). Efficiency of dogs was 4 times higher than human surveyors	Selection: calm disposition, physical stability, and persistence
				Reward: play
				Training: authors refer to published CDD literature
Savidge et al. (40)	Live reptile detection: brown tree snake	2 CDDs; 1 experienced and 1 inexp; 2 Retrievers	Accuracy of experienced CDD (44%) was higher than the inexperienced CDD (26%)	Selection: professional CDD organization
				Reward: play
				Training: narcotics, forensic, and SAR techniques
Smith et al. (13)	Scat detection: San Joaquin kit fox	7 CDDs; 2 Shepherds, 4 Retrievers, 1 mixed breed	Dogs correctly identified 100% of scats in scent line-up. Dogs correctly ignored incorrect scats 67% of the time	Selection: food or Play obsession
			In an uncontrolled field search dogs detected 0.43–5.37 scats/km	Reward: play/food
				Training: authors refer to published CDD and scent detection literature
Stevenson et al. (41)	Live reptile detection and shed skins: eastern indigo snake	1 CDD; experienced; mixed breed	Accuracy for live snakes was 81%, and for shed skins was 100%	Selection: professional CDD organization
				Reward: play
				Training: professional CDD organization
Vynne et al. (42)	Scat detection: 5 mammals	3 CDDs; <1 year experience	Dogs detected 2683 scats over 407 surveys. This was ~6.6 scats per dog team per search day. Detection rates varied between target species	Selection: play drive
				Reward: play
				Training: professional CDD organization. Authors refer to published CDD literature
Wasser et al. (14)	Scat detection: black and grizzly bear	9 CDDs; <1 year experience	Detection rates differed between years. Dogs detected 0.63–3.76 scats/ha in 1999, and detected 0.35–1.89 scats/ha in 2001	Selection: play drive/object orientation, temperament, trainability, and motivation
				Reward: play
				Training: narcotics, bomb, and arson detection, and search and rescue
Wasser et al. (43)	Scat detection: Spotted and Barred owl	2 CDDs; experienced; 2 mixed breeds	Accuracy of dogs was significantly higher than bird vocalization surveys. Ratio of dog surveys:vocalization surveys for northern spotted owl was 87:59%, and for Barred owl was 20.1:7.3%	Selection: high play drive. Authors also refer to published CDD literature
				Reward: play
				Training: authors refer to published CDD literature
Waters et al. (44)	Nest searches: bumble bee	1 CDD; <1 year experience; Spaniel	Accuracy of dog to detect known bumble bee nests was 100%	Selection: drug detection dog organization
			In uncontrolled tests dog found 33 nests	Reward: no mention
				Training: drug detection dog organization. Authors also refer to published CDD and scent detection literature
Wultsch et al. (45)	Scat detection: 5 native felines	1 CDD; <1 year experience	Dogs detected 1053 scats. 49% of these were identified to the species level	Selection: professional CDD organization
				Reward: no information
				Training: professional CDD organization

## Overview of Literature Search Results

As is evident from Table 1, current literature in the field primarily details what CDDs can do (4, 22, 30). The list is impressive! Detection of biological materials, such as scats, hair, urine, and burrows, makes up the majority of studies (76.7%), with studies focused solely on scat detection being most common of all (56.7%). Detection of live wildlife is reported in 20% of the studies, while 6.7% focus on carcass detection.

Of the studies in this review, 43.3% reported that dogs were acquired from professional CDD training organizations, or were selected using criteria developed by these organizations. A further 40% of studies reported adapting tools used for the selection and training of narcotics, explosives, SAR, cadaver, police, and forensics detection dogs. Of these, only one study reported on the specific assessment tools which were adapted (36). Noted in the review was that very few studies provided detailed information on the methods used to test or score specific characteristics, or how selection of traits may vary with respect to the external search environment or search target being selected for.

The lack of a standardized and publicly available assessment tool for CDDs, along with a widespread failure in available literature to report on specific selection processes, is a significant omission, particularly given that several studies show that CDD performance may be impacted by many factors. For example, environmental terrain/vegetation density (16), specific search target (42), and whether the target is terrestrial, arboreal, or marine (32, 39), all impact CDD effectiveness. Experience of the CDD team (40), handler characteristics (46), and the individuality of the dog selected (1, 20) are also important factors in CDD success.

When analyzing the specific characteristics commonly selected for in CDDs, it appeared that a strong play/food drive was the most common trait selected for. Appropriate temperament for the field of conservation detection was also cited often, and traits relating to problem solving, intelligence, and trainability were reportedly selected for in many studies. This suggests a considerable focus on psychological factors above biological or social factors and is important, because these traits are typically very poorly defined and difficult to measure. Biological characteristics of the dog, including agility, physical stability, and body type, were included as selection considerations in only a few studies in our review. This is not to say that biological traits are unimportant to selection considerations, however, as most studies which provided information on dog breed included at least one working or sporting dog, or a mixed breed. Thus, breed characteristics are likely to be important, even if this is not overtly stated. Only a few studies in the review considered the importance of sociability or dog-handler cooperation.

While existing literature rarely included sufficient information about selection, and about the reliability and validity of selection instruments, from our reading of the literature, we identified a number of biological, psychological, and social traits as being important. These are reviewed below. Biological traits included: morphology, including characteristics associated with the original working function of the dog’s breed, and olfactory, visual, and auditory acuity. Psychological traits included: personality; suitable levels of nerve strength; and drives, such as food, play, pack, and prey/hunt drives. Finally, social characteristics included: the possible importance of correctly pairing dog-handler teams based on the experience and psychological traits of both the dog and the handler.

## Biological Characteristics Likely to Affect Conservation Detection Dog Success

Several biological characteristics, grouped loosely into morphological characteristics and sensory capabilities, appear likely to be required for dogs to become successful CDDs. The perceived importance of morphological capabilities is not surprising since these dogs often work in challenging environmental landscapes (14, 16). Dogs working in SAR, cadaver, and disaster contexts may face similar challenges, which may explain why selection tools used in these contexts are frequently adapted for use with CDDs (36, 39, 44). The Brownell–Marsolais scale, for example, assesses morphological features (e.g., breed, size, health) and motor capabilities (e.g., agility, athleticism, and stamina) of candidate dogs.

### Morphology

Available studies show a strong trend toward using working and sporting dog breeds for conservation work, breeds with morphological characteristics which allow them to maintain performance in the face of challenging search environments, long working hours, and unfavorable weather conditions (16). Many of the dog breeds selected for CDD work are medium to large sized dogs (13, 20). These may be most suitable as CDDs, as dogs which are too large or small may struggle during extreme weather conditions (47). While Pugs, for example, appear capable of performing scent detection tasks to a similar standard to German Shepherd Dogs (48), they are not able to maintain body heat in extreme cold. It is also generally thought that smaller dogs with short, flat snouts have limited olfactory capabilities compared with their larger counterparts (49). At the other extreme, Great Danes may also be unsuited to this line of work, as their large size may make it difficult for them to cool down when working in strenuous environmental conditions or hot weather.

Several studies have suggested that the breed of dog (e.g., working or sporting dogs) (4), or even the genetic line of the dog, such as whether the dog comes from a working dog line or a show dog line, may be of particular importance (49). Dogs bred specifically for a working purpose have typically been selected for robust morphologies and, thus, may be more capable of withstanding the challenging field conditions associated with conservation detection work. Furthermore, medium sized dogs may be more capable of maneuvering in difficult environments, and may maintain stamina for longer search durations. This is not to say that small dogs do not have potentially important roles to play in conservation, particularly in environmental contexts with limited space. However, these should likely be selected from among working dog breeds, with careful consideration of their physical robustness. It may also be important to consider coat length and type. Dogs working in challenging terrains may require short coats or coats sufficient to provide thermal protection (50).

### Olfactory System

Olfaction is clearly a key sensory system for all scent detection dogs, regardless of context. Gazit and Terkel (47) demonstrated the importance of olfaction in a controlled environment when they showed that explosives dogs did not vary their search methods or detection success regardless of whether or not light conditions made the target visually obvious.

Selective breeding of dogs for scent work has resulted in some breeds of dog possessing several morphological features which facilitate their already exceptional olfactory capabilities. These include large ears and dewlaps to catch scent particles, and wide, elongated noses with a large nasal cavity to house the olfactory epithelium and increase the number of odor receptor cells (ORCs), all of which aid in scent detection capabilities. Dog breeds differ in the number of ORCs they possess. Bloodhounds possess the largest number of ORCs of any dog breed, with 300 million. However, Bloodhounds are not commonly used in conservation detection work (4). Working and sporting dogs, such as Shepherds, Retrievers, Collies, and Setters, are more common (4) despite having fewer ORCs (e.g., Shepherds have around 225 million ORCs).

An individual’s olfactory sensitivity alone, then, is evidently not the only relevant characteristic for this type of work. While one might presume that a number of ORCs above some theoretical threshold level is required, many other traits also determine success. In this respect, it is instructive to consider the type of scent being detected. In scent detection work there are three search types that a dog can perform: air-scenting, in which the nose of the dog is held in the air “sniffing” to catch scent on the wind; tracking, where the nose is held close to the ground, following the scent and direction of the target; and, trailing, in which dogs use a combination of air-scenting and tracking techniques (51, 52).

Air-scenting is the most common search technique used in conservation detection, and may be what enables CDDs to cope with the often challenging nature and long working hours of the search environment. Early work by Steen et al. (53) suggested that game hunting dogs are capable of maintaining strong olfactory capabilities while working in challenging environments, under strenuous physiological conditions. This ability appears to be due to the Bernoulli effect, which occurs when the dog runs at a high speed with its nose held up, “sniffing” into the air. Craven et al. (54) later determined that canine olfactory acuity should be most effective during sniffing. During sniffing each nostril acquires spatially separate odor samples (known as “bilateral scent intake”). Fluid dynamics within the nose increase aerodynamic flow of the odor sample, creating an active aerodynamic sampling system which is specialized for odor detection and discrimination (54, 55). Due to this discrimination function, it is likely that dogs which perform air-scenting are able to detect airborne scents in large area searches where there is no scent trail to follow.

There is extensive literature on the topics of tracking and trailing dogs (56), and the capabilities of scent detection dogs in game hunting, SAR, and several other high intensity search environments (52). However, there is very little information on how dogs maintain strong scenting abilities in varying environmental conditions, and practically no literature detailing the possible impacts of choosing a natural air-scenting dog breed (e.g., Spaniels) for conservation work (57). Currently available assessment tools used for scent detection dogs do not appear to account for the dogs’ natural preference toward one type of scenting work.

### Visual and Auditory Systems

Hurt and Smith (22) suggest that the specific auditory and visual acuity required of scent detection dogs may vary between the differing scent detection fields, and even within fields, based on the nature of the search environment and specific target of interest. Polgár et al. (58) have since shown that, while pet dogs are capable of using olfactory cues alone to make correct decisions in a basic choice task, they will often prioritize the use of other search strategies. CDDs should demonstrate a baseline level of visual acuity to aid them in searching complex environments (6). Greater visual acuity may also aid in detection of live wildlife (2). This is particularly likely in situations where environmental conditions, such as wind strength, are unfavorable, allowing the dog to locate movement in the distance, before honing in on the target’s exact location using olfactory cues. However, for detection of stationary targets, such as scats and plants, it has been suggested that dogs do not rely heavily on visual acuity to assist in detection. Goodwin et al. (15) found that, in detection of the Spotted knapweed plant (*Centeurea maculosa*), human surveyors were more successful in locating large targets than small targets, whereas dogs were similarly successful in locating small and large targets. Brook et al. (29) also showed that, while dogs were effective at locating the area in which Javan rhinoceros (*Rhinoceros sondaicus*) dung was located, human surveyors were much faster than dogs at visually locating dung. It was suggested that, even when rhinoceros dung was visible, dogs still relied predominantly on olfaction to locate the target.

We could find no information regarding the importance of auditory acuity in conservation detection. However, Brownell and Marsolais (21) suggest that, for dogs working in SAR/disaster detection, auditory acuity should be relatively strong. This likely reflects the search environment which, similar to conservation detection environments, requires strong dog-handler communication (22, 46) to facilitate efficient searching in ever-changing environmental conditions (13). Several tools developed for other fields of scent detection, such as the United States Department of Agriculture (USDA) National Detector Dog scale (59), provide scoring systems to measure a dog’s physical health, including a section to measure sensory systems. However, we could find no studies examining whether scent dog performance in any context varied with auditory acuity. Perhaps, as with olfactory ability, it is presumed that any dog with visual and auditory acuity above some theoretical baseline measure will be able to perform satisfactorily. Individual differences in sensory traits are not evaluated, much less reported upon, in available literature.

### Summary of Important Biological Characteristics

From this brief review, we conclude that there are several biological traits which should be considered when selecting dogs for conservation detection. These include morphological characteristics, often associated with the original working function of the dogs’ breed and olfactory, visual, and auditory capabilities. Because these biological traits are equally important across several scent detection disciplines, established tools for the assessment of scent detection dogs in other contexts should provide sufficient information regarding the types of biological traits to be measured and how this is best accomplished. This is indeed the case for the USDA National Detector Dog scale (59), which measures physical soundness. More specialized tests which account for the specific nature of the search environment and target scent should also be considered. SAR detection contexts are perhaps the most similar fields in terms of environmental search habitat; however, the desired biological traits may differ between these fields and environmental contexts due to differences in scenting types required based on the types of targets being detected.

## Psychological Characteristics Likely to Affect Conservation Detection Dog Success

While physical traits reflecting biological characteristics are undoubtedly important in determining the ability of a dog to work as a CDD, taken alone they are insufficient to ensure success. Temperament, personality, and behavior also play a critical role in determining CDD success or failure (2, 4). According to Ley and Bennett (60), *temperament* is a generalized behavioral style, present from birth and typically reflecting a genetic predisposition, while *personality* is functionally indistinguishable from temperament, but is the result of both the dog’s genetics and experiences over time, which together shape the dog’s characteristic style of responding to situational events. *Behavior*, meanwhile, is how a dog responds at any given moment to a situation (60). While behavior normally reflects underlying personality traits, it is also partially determined by situational factors.

Individual psychological differences between dogs are thought to influence performance in working tasks, even in dogs with similar biological traits (4, 61). Several reports in the scientific literature suggest the importance of considering specific psychological traits in potential CDDs (1, 4). The strongest evidence for this comes from studies where individual differences are pronounced, even when physiological and environmental factors are controlled for. O’Connor et al. (20) showed marked variability between two dogs trained to detect bumble bee nests in controlled scent line ups (68%: 100%). Another study by Kerley and Salkina (19) showed individual differences between five experienced CDDs, asked to detect and differentiate between the scat of Amur Tigers. While four of these dogs progressively became more proficient with repeated trials (87–98% accuracy over time), it was unclear why the fifth dog did not show similar improvements. It is possible that psychological factors, such as personality characteristics, nerve strength, and motivational drives, may have had some impact on these outcomes.

### Personality Characteristics

One of the main difficulties faced by behavioral researchers, and indeed one of the difficulties we faced in reviewing the scientific literature on conservation detection, is that many different words may be used to describe very similar behavioral traits. This makes it difficult to pinpoint exactly which traits are of key importance to CDD success. To address this problem, several researchers have proposed using discrete categories to encompass a broad spectrum of temperament, personality, and behavioral traits (61–63). For example, Jones and Gosling (63) proposed that most psychological dimensions fit within seven broad temperament categories: reactivity/excitability–stability; fearfulness–courage/confidence; aggression–agreeableness; sociability/friendliness–lack of interest in others; responsiveness to training; dominance–submission; activity level.

Prior to the detailed conceptualization of behavioral traits provided by Jones and Gosling (63); Svartberg (61) determined that a boldness–shyness index could act as an umbrella variable under which several psychological traits predictive of success in working dogs could fit. They included sociability toward strangers, playfulness, interest to chase, exploration, and fearlessness as evidence of boldness. The scientific literature suggests that boldness may be a particularly important trait in successful completion of working tasks. This is because individuals with low boldness scores are more fearful, anxious, and easily distracted (61). Thus, they take longer and often require different training methods to become successful in working tasks, or may never be successful despite intensive training.

### Nerve Strength

Many tools which are commonly used for assessment of scent detection dogs assess something called “nerve strength.” This is perhaps associated with boldness in that it describes the behavioral response of a dog to an unfamiliar stimulus (e.g., tactile, auditory, or visual stimulus) at a point in time. The Brownell–Marsolais (21) scale, for example, which is used for SAR and disaster detection dogs, measures an individuals’ behavioral responses to unfamiliar stimuli. Behaviors exhibited by the dog, such as fearfulness, curiosity, or anxiety, provide an indication of the dog’s nerve strength, indicating to researchers the suitability of the dog for particular fields of scent detection.

Of particular relevance to conservation detection is tactile nerve strength, which measures a dog’s response to unfamiliar surfaces and spaces. Auditory nerve strength, meanwhile, measures the dog’s reactivity to unexpected/unfamiliar noises. Behaviors exhibited during nerve strength assessments may be suggestive of a dog’s ability to work efficiently in unfamiliar search areas (also referred to as environmental stability). Rolland et al. (39) demonstrated the importance of tactile nerve strength for dogs working on boats to detect floating feces of marine mammals. Hurt and Smith (22) also suggest the importance of good tactile nerve strength due to the complex, highly variable nature of the search environment typical of conservation detection. They note, however, that nerve strength may be less important to dogs working in a conservation context than it would be to military/police dogs, due to the nature of the work and search environment (22).

Tests which measure nerve strength in potential CDDs may be adapted from those used to assess SAR, disaster, and cadaver dogs due to similarities in the search environments. However, the environments CDDs may work in are particularly diverse and, as yet, no standardized assessment exists to measure visual, auditory, or tactile nerve strength in potential CDDs. Development of a standardized tool to assess these psychological characteristics may be particularly important to conservation detection, as it may aid in assessing behaviors appropriate to each specific field of conservation detection.

### Motivational Drives

Several authors have also stressed the importance of object obsession and strong drives, such as food and play drives, as contributors to the potential success of CDDs (14, 36). This is evidenced by the fact that, of the studies in Table 1 which provided information on CDD selection, 80% stated food or play drives as selection criteria. These psychological factors are likely to be of great importance, but it is exceedingly difficult to operationalize what drives are and how they can be elicited in dogs in a way that can be reliably measured. In this review, we use the definitions for drives which were established by Brownell and Marsolais (21), who divided drives into six categories: pack/social; play; food; prey; hunt; and defense.

Brownell and Marsolais (21) proposed that drive-testing should be a critical part of the assessment process when selecting a dog for disaster/SAR scent work, as drives are thought to be strongly associated with sustained search intensity and trainability. The similar working requirements of SAR dogs and CDDs, including the requirement for sustained working motivation over several hours in challenging environments, suggests that the Brownell–Marsolais drive test may be applicable to selection of dogs for conservation scent detection. However, the unique ecosystems in which CDDs are sometimes required to work may mean that the existing test needs to be modified for use in this context.

Clear benefits of selecting dogs which demonstrate excessive food/play drives for scent detection roles have consistently been reported in the scientific literature. Work on the selection and training of narcotics dogs by Maejima et al. (64), for example, and selection of cadaver detection dogs by Dorriety (65) has confirmed that object obsession is associated with decreased distractibility and increased trainability, which should, in turn, be associated with scent detection dog success (64, 65). One reason why strong object/food obsession may be so important is that scent detection dog training requires the trainer to associate the target scent with an object of intrinsic pleasure for the dog, such as a ball or a food reward (66, 67). The more the dog desires the ball or food, the more successful the training process is, since this desire increases the dog’s focus, decreases distractibility, and increases motivation to work for sustained periods of time (68).

Several studies documenting success in field studies by CDDs have stated that dogs were initially chosen on the basis of strong play or chase drives (14, 36). To the best of our knowledge, however, no studies have provided quantitative, laboratory-based evidence demonstrating the importance of specific drives for CDD success (22). Future research which documents learning times, success rates, and efficacy of current training methods for dogs which vary in these drives would be beneficial. This is especially relevant in the case of community run CDD programs, where there may be fewer options to reject dogs based on low drive, due to a dependence on volunteers. More research is needed to examine the extent to which drives are both necessary and sufficient for success. If found to be necessary, then strategies for identifying and quantifying specific drives at an early age require development.

The importance of food/play drive is thought applicable across all fields of scent detection, as it facilitates scent detection training and sustained motivation during searching. Other drives, however, including prey, hunt, and search drives, have also been related to sustained search motivation during scent detection tasks in various contexts (2). The applicability of these drives in the specific context of conservation work remains less certain. Cablk and Heaton (31) mention that dogs with high prey drive may be unsuited to live wildlife detection, due to the potential for harm to be caused to the target species. Dogs working in natural ecosystems may cause harm to the wildlife they are employed to detect, and may be too easily distracted by non-target scents. Hurt and Smith (22) similarly suggest that strong prey/hunt drives may be detrimental to conservation detection, although they also asserted that strong search drives should aid in sustained attention to the working task. This could present a problem to end users wishing to utilize existing assessment tools to identify potential CDDs, as desired levels of prey, hunt, and search drives are not well defined or addressed in existing tools. It is also not known whether good training can overcome any potential for high prey drive to be detrimental.

Another psychological characteristic of interest in this context is sociability or pack drive, the ability of a dog to cooperate with a handler, and to work in the presence of unfamiliar dogs or humans without exhibiting anxiety, fearfulness, or aggression. The importance of a moderate to high sociability/pack drive in fields such as biosecurity or narcotics detection is well established and reflects the human-oriented nature of the search environment, which requires that dogs exhibit specific, predictable behaviors around unfamiliar people. In contrast, the specific search environment of conservation detection is less focused on the dog’s response to unfamiliar people, but may necessitate a moderate to high level of sociability/pack drive to facilitate cooperation with a human handler. The Brownell–Marsolais scales assess directability in potential dogs, which is likely to be applicable to CDDs. This is difficult to conclude with any certainty, however, as the perceived importance of sociability appears to vary between organizations and with respect to the specific search environment and target.

### Summary of Psychological Characteristics

In summary, our review of the scientific literature suggests that many psychological traits are likely to impact on CDD success. Strong motivation and obsessive food/play drives are considered important in a large proportion of the scientific literature, although there is little evidence for whether one drive might be better than another in specific contexts. This may well vary according to the specific nature of conservation detection work being performed, but there is no way of assessing this based on existing tools. Dahlgren et al. (4) have suggested the importance of considering a dog breed’s original working function, as this may make specific psychological characteristics more pronounced. For example, a Setter may be more skilled at detecting a live bird than a Shepherd, but may also have a higher hunt/chase drive (4). Individual variability within dog breeds is pronounced, however, and we would caution against selecting dogs purely on the basis of their breed heritage.

## Social Characteristics Likely to Affect Conservation Detection Dog Success

Dogs and humans have shared a close social relationship for thousands of years (69). This has resulted in the ability of humans and dogs to successfully interact in modern societies, governed by shared rules (70). Most studies involving CDDs have focused on the ability of dogs to find specific targets, and there is little doubt that more attention should be paid to other factors affecting their success. These include the dog-handler team and, specifically, the contested importance of the relationship between dog and handler in contributing to CDD success (22, 46, 71). In this section, we briefly review features of dogs’ social intelligence, and discuss the likely contribution of dog sociability to the potential success of CDDs.

### Social Intelligence

Over recent years, dog cognition research has increased, with several studies focusing specifically on dog-handler relationships and inter-species cooperation (72). Miklósi et al. (73) demonstrated a strong social intelligence in dogs by showing that dogs were able to interpret human hand gestures and subtle changes in body positioning to find hidden food in a manner similar to young children. Gácsi et al. (74) suggested that the success of dogs in recognizing subtle human communicative gestures is the result of selection for attention to, and cooperation with, humans.

We could find no studies specifically investigating how variability in canine social intelligence may affect the success of scent detection dogs, although it is instructive that experienced CDDs appear capable of working successfully with unfamiliar handlers to a sufficient operational standard. Brook et al. (29) successfully located Javan rhinoceros (*R. sondaicus*) dung using two professional CDDs, after just 3 weeks of handler training and dog acclimation to the new handler and new working environment. Another study, by Dematteo et al. (18), demonstrated the efficacy of professionally trained CDDs to work with an unfamiliar handler. These dogs successfully located the presence of bush dogs (*Speothos venaticus*) after only 2 weeks of handler training.

### Handler Characteristics

While a growing body of literature shows that some CDDs can work with multiple or unfamiliar handlers (18, 29), we could find no studies in which the same dog’s performance was compared when the dog was handled by a familiar versus unfamiliar, but equally experienced, handler. Hence, it is unclear whether these dogs would be more successful in their respective fields when paired with a familiar handler with experience in reading both the dog and the environment. It is also unclear whether a certain level of dog handling expertise is required before undertaking the training necessary to specifically handle CDDs, although the Scientific Working Group on Dog and Orthogonal detector Guidelines (SWGDOG) provide detailed recommendations concerning initial selection and training of scent dog handlers (75).

It is possible that handler experience may be particularly important for CDDs, due to the complex nature of working in unpredictable and constantly changing search environments. Furtado et al. (71) described a study which demonstrated an inexperienced handler effect during Jaguar (*Panthera onca*) scat detection. In this study, two experienced dog-handler teams had an 81% accuracy rate when collecting Jaguar and Puma scats, while an inexperienced dog-handler team collected 50% non-target species. This can be problematic, as misidentification of scats in the field may mean that inexperienced handlers reward incorrect dog behavior. On this basis, further research is needed into the effects of handler experience or handler characteristics on CDD outcomes. Inexperienced handlers may be less capable of directing dog searches in accordance with environmental conditions, and may be more likely to reward dogs too early or on the wrong scent, inadvertently reducing detection accuracy and success (71). Furthermore, dogs which do not demonstrate a strong sociability/pack drive may require a more confident/experienced handler to ensure cooperation between the dog-handler team.

### Summary of Social Characteristics

It appears that little is known regarding how social characteristics of dogs can impact on search success. Yet, this is a critical issue, since the nature of conservation work requires close cooperation between dog and handler. As a species, dogs are highly competent in understanding humans, but individual and breed-based differences are likely to impact performance. These have not yet been investigated or documented. Handler characteristics, particularly their level of expertise, are also likely to be of critical importance, as is the nature of the relationship between an individual dog and his or her handler. The framework established by the BPS model may act as a tool to help us better understand how interactions between a dog’s social environment and its psychological and biological traits may act together to influence performance, but conclusions regarding this are not yet supported by available literature.

## Conclusion and Recommendations

Many of the characteristics required in potential CDD candidates are similar to those assessed by instruments developed for selection of dogs in other contexts, such as narcotics, explosives, cadaver, forensics, and SAR detection dogs. This is to be expected since conservation detection shares many commonalities with these other fields of scent detection. However, many of the traits are psychological and social rather than physical, and this makes it difficult for them to be accurately assessed, particularly by novices. While several articles we reviewed reported adapting selection tools from other fields of scent detection, most gave little or no information regarding which tools were used, how they were adapted, or the specific selection criteria used to assess CDD candidates.

A lack of available information regarding the selection process is likely to make it extremely difficult for conservation groups to identify suitable dogs, upon which scarce resources can then be expended for training and deployment purposes. This, we believe, represents a substantial barrier to the wider use of CDDs. While any group of volunteers can acquire and utilize other resources for tracking endangered animals or detecting invasive plants, only those with access to already trained dogs, or to somebody who already possesses the skills required to select and train dogs, are likely to be able to benefit from the impressive skills appropriately selected and trained dogs demonstrate. We therefore urge those working in this field to be generous in providing access to information regarding selection of dogs, and also rigorous in documenting and empirically justifying their selection processes. We also urge those considering acquiring their first “potential” CDD to consult with experts first about the biological, psychological, and social characteristics most likely to predict success. Adapting existing tools which assess characteristics thought to be applicable across all fields of conservation detection (e.g., traits such as robust morphology, play/food drive, directability, and social cooperation) is likely to be the first step in developing a standardized assessment for CDDs. Unique requirements of the role, however, mean that careful adaptation is likely to be required.

## Author Contributions

SB conducted the literature searches and wrote the first draft of the manuscript. TH worked with SB to structure and edit the content. PB played a leadership role in devising and setting the scope for the manuscript.

## Conflict of Interest Statement

The authors declare that the research was conducted in the absence of any commercial or financial relationships that could be construed as a potential conflict of interest.

## References

[1] Robert M Laporte P Field techniques for studying breeding Yellow Rails. J Field Ornithol (1994) 68(1):56–63.

[2] Helton WS Introduction to the new science of working dogs. In: Helton WS, editor. Canine Ergonomics: The Science of Working Dogs. Boca Raton, FL: CRC Press (2009). p. 1–15.

[3] Jenkins D Watson A Miller GR Population studies on red grouse, *Lagopus lagopus scoticus* (Lath.) in north-east Scotland. J Anim Ecol (1963) 32(3):317–76. 10.2307/2598

[4] Dahlgren DK Elmore RD Smith DA Hurt A Arnett EB Connelly JW Use of dogs in wildlife research and management. 7th ed In: Silvy NJ, editor. The Wildlife Techniques Manual Volume 1. Baltimore, ML: The John Hopkins University Press (2012). p. 140–53.

[5] Gutzwiller K Minimizing dog-induced biases in game bird research. Wildl Soc Bull (1990) 18(3):351–6.

[6] Long RA Donovan TM Mackay P Zielinski WJ Buzas JS Comparing scat detection dogs, cameras, and hair snares for surveying carnivores. J Wildl Manage (2007) 71(6):2018–25. 10.2193/2006-292

[7] Nussear KE Esque TC Heaton JS Cablk ME Drake KK Valentin C Are wildlife detector dogs or people better at finding desert tortoises (*Gopherus Agassizii*)? J Herpetol Conserv Biol (2008) 3(1):103–15.

[8] Duggan JM Heske EJ Schooley RL Hurt A Whitelaw A Comparing detection dog and livetrapping surveys for a cryptic rodent. J Wildl Manag (2011) 75(5):1209–17. 10.1002/jwmg.150

[9] Homan HJ Linz G Peer BD Dogs increase recovery of passerine carcasses in dense vegetation. Wildl Soc Bull (2001) 29(1):292–6. 10.2307/3784011

[10] Arnett EB A preliminary evaluation on the use of dogs to recover bat fatalities at wind energy facilities. Wildl Soc Bull (2006) 34(5):1440–5. 10.2193/0091-7648(2006)34[1440:APEOTU]2.0.CO;2

[11] Paula J Leal MC Silva MJ Mascarenhas R Costa H Mascarenhas M Dogs as a tool to improve bird-strike mortality estimates at wind farms. J Nat Conserv (2011) 19(4):202–8. 10.1016/j.jnc.2011.01.002

[12] Mathews F Swindells M Goodhead R August TA Hardman P Linton DM Effectiveness of search dogs compared with human observers in locating bat carcasses at wind-turbine sites: a blinded randomized trial. Wildl Soc Bull (2013) 37(1):34–40. 10.1002/wsb.256

[13] Smith DA Ralls K Hurt A Adams B Parker M Davenport B Detection and accuracy rates of dogs trained to find scats of San Joaquin kit foxes (*Vulpes macrotis mutica*). Anim Conserv (2003) 6(4):339–46. 10.1017/S136794300300341X

[14] Wasser SK Davenport B Ramage ER Hunt KE Parker M Clarke C Scat detection dogs in wildlife research and management: application to grizzly and black bears in the Yellowhead Ecosystem, Alberta, Canada. Can J Zool (2004) 82(3):475–92. 10.1139/Z04-020

[15] Goodwin KM Engel RE Weaver DK Trained dogs outperform human surveyors in the detection of rare spotted knapweed (*Centaurea stoebe*). Invasive Plant Sci Manag (2010) 3(2):113–21. 10.1614/IPSM-D-09-00025.1

[16] Leigh KA Dominick M An assessment of the effects of habitat structure on the scat finding performance of a wildlife detection dog. Methods Ecol Evol (2015) 6(7):745–54. 10.1111/2041-210X.12374

[17] Harrison RL A comparison of survey methods for detecting bobcats. Wildl Soc Bull (2006) 34(2):548–52. 10.2193/0091-7648(2006)34[548:ACOSMF]2.0.CO;2

[18] Dematteo KE Rinas MA Sede MM Davenport B Argüelles CF Lovett K Detection dogs: an effective technique for bush dog surveys. J Wildl Manag (2009) 73(8):1436–40. 10.2193/2008-545

[19] Kerley LL Salkina GP Using scent-matching dogs to identify individual Amur Tigers from scats. J Wildl Manage (2007) 71(4):1349–56. 10.2193/2006-361

[20] O’Connor S Park KJ Goulson D Humans versus dogs; a comparison of methods for the detection of bumble bee nests. J Apic Res (2012) 51(2):204–11. 10.3896/IBRA.1.51.2.09

[21] Brownell DA Marsolais M The Brownell-Marsolais scale: a proposal for the qualitative evaluation of SAR/disaster K9 candidates. Adv Rescue Technol (2002):57–67. Available from: www.operationtakemehome.org/sar/Canine%20Downloads/Forms/AllItems.aspx

[22] Hurt A Smith DA Conservation dogs. In: Helton WS, editor. Canine Ergonomics: The Science of Working Dogs. Boca Raton, FL: CRC Press (2009). p. 175–94.

[23] Engel GL. The need for a new medical model: a challenge for biomedicine. Science (1977) 196(4286):129–36. 10.1126/science.847460 847460

[24] Ghaemi SN The biopsychosocial model in psychiatry: a critique. Existenz (2011) 6(1):1–8.

[25] Olson CM Strawderman MS. Modifiable behavioral factors in a biopsychosocial model predict inadequate and excessive gestational weight gain. J Am Diet Assoc (2003) 103(1):48–54. 10.1053/jada.2003.50001 12525793

[26] Griffiths MD A ‘components’ model of addiction within a biopsychosocial framework. J Subst Use (2005) 10(4):191–7. 10.1080/14659890500114359

[27] Schotte CKW Van Den Bossche B De Doncker D Claes S Cosyns P. A biopsychosocial model as a guide for psychoeducation and treatment of depression. Depress Anxiety (2006) 23:312–24. 10.1002/da.20177 16688730

[28] Arandjelovic M Bergl RA Ikfuingei R Jameson C Parker M Vigilant L. Detection dog efficacy for collecting faecal samples from the critically endangered Cross River gorilla (*Gorilla gorilla diehli*) for genetic censusing. R Soc Open Sci (2015) 2:140423. 10.1098/rsos.140423 26064602 PMC4448817

[29] Brook SM van Coeverden de Groot P Scott C Boag P Long B Ley RE Integrated and novel survey methods for rhinoceros populations confirm the extinction of *Rhinoceros sondaicus annamiticus* from Vietnam. Biol Conserv (2012) 155:59–67. 10.1016/j.biocon.2012.06.008

[30] Browne CM Stafford KJ Fordham RA The detection and identification of tuatara and gecko scents by dogs. J Vet Behav (2015) 10(6):496–503. 10.1016/j.jveb.2015.08.002

[31] Cablk ME Heaton JS. Accuracy and reliability of dogs in surveying for desert tortoise (*Gopherus agassizii*). Ecol Appl (2006) 16(5):1926–35. 10.1890/1051-0761(2006)016[1926:AARODI]2.0.CO;2 17069383

[32] Chambers CL Vojta CD Mering ED Davenport B Efficacy of scent-detection dogs for locating bat roosts in trees and snags. Wildl Soc Bull (2015) 39(4):780–7. 10.1002/wsb.598

[33] Cristescu RH Foley E Markula A Jackson G Jones D Frère C. Accuracy and efficiency of detection dogs: a powerful new tool for koala conservation and management. Sci Rep (2015) 5:8349. 10.1038/srep08349 25666691 PMC4322364

[34] Hagell SE Conserving Forest Connectivity for the Central American Spider Monkey (Ateles geoffroyi) in Southwestern Nicaragua [Dissertation]. Flagstaff, AZ: Northern Arizona University (2010).

[35] Oliveira ML de Norris D Ramírez JFM Peres PH de F Galetti M Dogs can detect scat samples more efficiently than humans: an experiment in a continuous Atlantic Forest remnant. Zoologia (2012) 29(2):183–6. 10.1590/s1984-46702012000200012

[36] Reed SE Bidlack AL Hurt A Getz WM Detection distance and environmental factors in conservation detection dog surveys. J Wildl Manage (2011) 75(1):243–51. 10.1002/jwmg.8

[37] Reindl SA Efficacy of Scent Dogs in Detecting Black-Footed Ferrets (Mustela nigripes) at a Reintroduction Site in South Dakota [Dissertation]. Dakota, USA: South Dakota State University (2004).

[38] Robertson HA Fraser JR Use of trained dogs to determine the age structure and conservation status of kiwi *Apteryx* spp. populations. Bird Conserv Int (2009) 19:121–9. 10.1017/S0959270908007673

[39] Rolland RM Hamilton PK Kraus SD Davenport B Gillett RM Wasser SK Faecal sampling using detection dogs to study reproduction and health in North Atlantic right whales (*Eubalaena glacialis*). J Cetacean Res Manag (2006) 8(2):121–5.

[40] Savidge JA Stanford JW Reed RN Haddock GR Adams AAY Canine detection of free-ranging brown treesnakes on Guam. N Z J Ecol (2010) 35(2):174–81.

[41] Stevenson DJ Ravenscroft KR Zappalorti RT Ravenscroft MD Weigley SW Jenkins CL Using a wildlife detector dog for locating Eastern Indigo snakes (*Drymarchon couperi*). Herpetol Rev (2010) 41(4):437–42.

[42] Vynne C Skalski JR Machado RB Groom MJ Jácomo ATA Marinho-Filho J Effectiveness of scat-detection dogs in determining species presence in a tropical savanna landscape. Conserv Biol (2010) 25(1):154–62. 10.1111/j.1523-1739.2010.01581.x 21029162

[43] Wasser SK Hayward LS Hartman J Booth RK Broms K Berg J Using detection dogs to conduct simultaneous surveys of northern spotted (*Strix occidentalis caurina*) and barred owls (*Strix varia*). PLoS One (2012) 7(8):e42892. 10.1371/journal.pone.0042892 22916175 PMC3419739

[44] Waters J O’Connor S Park K Goulson D Testing a detection dog to locate bumblebee colonies and estimate nest density. Apidologie (2010) 42(2):200–5. 10.1051/apido/2010056

[45] Wultsch C Waits LP Kelly MJ. Noninvasive individual and species identification of jaguars (*Panthera onca*), pumas (*Puma concolor*) and ocelots (*Leopardus pardalis*) in Belize, Central America using cross-species microsatellites and faecal DNA. Mol Ecol Resour (2014) 14(6):1171–82. 10.1111/1755-0998.12266 24751217

[46] Bennett E Observations from the use of dogs to undertake carcass searches at wind facilities in Australia. In: Hull C Bennett E Stark E Smales I Lau J Venosta M, editors. Wind and Wildlife: Proceedings from the Conference on Wind Energy and Wildlife Impacts, October 2012, Melbourne Australia Netherlands: Springer Science (2015). p. 113–23.

[47] Gazit I Terkel J Domination of olfaction over vision in explosives detection by dogs. Appl Anim Behav Sci (2003) 82(1):65–73. 10.1016/S0168-1591(03)00051-0

[48] Hall NJ Glenn K Smith DW Wynne CDL Performance of pugs, German shepherds, and greyhounds (*Canis lupus familiaris*) on an odor-discrimination task. J Comp Psychol (2015) 129(3):237–46. 10.1037/a0039271 26010195

[49] Polgár Z Kinnunen M Ujváry D Miklósi A Gácsi M. A test of canine olfactory capacity: comparing various dog breeds and wolves in a natural detection task. PLoS One (2016) 11(5):e0154087. 10.1371/journal.pone.0154087 27152412 PMC4859551

[50] Bulanda S Ready! Training the Search and Rescue Dog. 2nd ed Freehold, NJ: Kennel Club Books (2010).

[51] Syrotuck WG Scent and the Scenting Dog. Mechanicsburg, PA: Barkleigh Productions (1972).

[52] Jones KE Dashfield K Dowend AB Otto CM Search-and-rescue dogs: an overview for veterinarians. J Am Vet Med Assoc (2004) 225(6):854–60. 10.2460/javma.2004.225.854 15485043

[53] Steen JB Mohus I Kvesetberg T Walløe L. Olfaction in bird dogs during hunting. Acta Physiol Scand (1996) 157:115–9. 10.1046/j.1365-201X.1996.479227000.x 8735662

[54] Craven BA Paterson EG Settles GS The fluid dynamics of canine olfaction: unique nasal airflow patterns as an explanation of macrosmia. J R Soc Interface (2009) 7(47):933–43. 10.1098/rsif.2009.0490 20007171 PMC2871809

[55] Settles GS Kester DA Dodson-Dreibelbis LJ The external aerodynamics of canine olfaction. In: Bath FG Humphrey JAC Secomb TW, editors. Sensors and Sensing in Biology and Engineering. Vienna, Austria: Springer-Verlag (2003). p. 323–35.

[56] Wells DL Hepper PG Directional tracking in the domestic dog, *Canis familiaris*. Appl Anim Behav Sci (2003) 84(4):297–305. 10.1016/j.applanim.2003.08.009

[57] Bradshaw JWS Jackson SL Changes in rates of sniffing in air-trailing dogs. J Vet Behav (2009) 4(6):253–4. 10.1016/j.jveb.2009.06.005

[58] Polgár Z Miklósi A Gácsi M. Strategies used by pet dogs for solving olfaction-based problems at various distances. PLoS One (2015) 10(7):e0131610. 10.1371/journal.pone.0131610 26176609 PMC4503413

[59] USDA Animal and Plant Health Inspection Service. USDA National Detector Dog Manual. (2012). Available from: www.aphis.usda.gov/import_export/plants/manuals/ports/downloads/detector_dog.pdf

[60] Ley JM Bennett PC Understanding personality by understanding companion dogs. Anthrozoös (2007) 20(2):113–24. 10.2752/175303707X207909

[61] Svartberg K Shyness–boldness predicts performance in working dogs. Appl Anim Behav Sci (2002) 79(2):157–74. 10.1016/S0168-1591(02)00120-X

[62] Rooney NJ Bradshaw JW Breed and sex differences in the behavioural attributes of specialist search dogs – a questionnaire survey of trainers and handlers. Appl Anim Behav Sci (2004) 86(1):123–35. 10.1016/j.applanim.2003.12.007 15027550

[63] Jones AC Gosling SD Temperament and personality in dogs (*Canis familiaris*): a review and evaluation of past research. Appl Anim Behav Sci (2005) 95(1–2):1–53. 10.1016/j.applanim.2005.04.008

[64] Maejima M Inoue-Murayama M Tonosaki K Matsuura N Kato S Saito Y Traits and genotypes may predict the successful training of drug detection dogs. Appl Anim Behav Sci (2007) 107(3–4):287–98. 10.1016/j.applanim.2006.10.005

[65] Dorriety JK Cadaver dogs as a forensic tool: an analysis of prior studies. J Forensic Ident (2007) 57(5):717–25.

[66] Panksepp J Biven L The Archaeology of Mind: Neuroevolutionary Origins of Human Emotions. New York: WW Norton & Company (2012).

[67] Berridge KC Kringelbach ML. Neuroscience of affect: brain mechanisms of pleasure and displeasure. Curr Opin Neurobiol (2013) 23(3):294–303. 10.1016/j.conb.2013.01.017 23375169 PMC3644539

[68] Christiansen FO Bakken M Braastad B. Behavioural differences between three breed groups of hunting dogs confronted with domestic sheep. Appl Anim Behav Sci (2001) 72(2):115–29. 10.1016/S0168-1591(00)00202-1 11278031

[69] Morey DF Burying key evidence: the social bond between dogs and people. J Archaeol Sci (2006) 33(2):158–75. 10.1016/j.jas.2005.07.009

[70] Hare B Tomasello M. Human-like social skills in dogs? Trends Cogn Sci (2005) 9(9):439–44. 10.1016/j.tics.2005.07.003 16061417

[71] Furtado MM Carrillo-Percastegui SE Jácomo ATA Powell G Silveira L Vynne C Studying Jaguars in the wild: past experiences and future perspectives. CAT News. Special Issue 4 – The Jaguar in Brazil. (2008). p. 41–7. Available from: http://www.catsg.org/index.php?id=196

[72] Topál J Gácsi M Miklósi A Virányi Z Kubinyi E Csányi V Attachment to humans: a comparative study on hand-reared wolves and differently socialized dog puppies. Anim Behav (2005) 70:1367–75. 10.1016/j.anbehav.2005.03.025

[73] Miklósi A Kubinyi E Topál J Gácsi M Virányi Z Csányi V A simple reason for a big difference: wolves do not look back at humans, but dogs do. Curr Biol (2001) 13(9):763–6. 10.1016/S0960-9822(03)00263-X 12725735

[74] Gácsi M Gyoöri B Virányi Z Kubinyi E Range F Belényi B Explaining dog wolf differences in utilizing human pointing gestures: selection for synergistic shifts in the development of some social skills. PLoS One (2009) 4(8):e6584. 10.1371/journal.pone.0006584 19714197 PMC2719091

[75] SWGDOG. Scientific Working Group on Dog and Orthogonal Detector Guidelines SC5 – Selection of Handlers (2006). Posted Under Approved Guidelines. (2006). Available from: http://swgdog.fiu.edu/

